# Re-Evaluating the Possible Increased Risk of HIV Acquisition With Progestin-Only Injectables Versus Maternal Mortality and Life Expectancy in Africa: A Decision Analysis

**DOI:** 10.9745/GHSP-D-17-00243

**Published:** 2017-12-28

**Authors:** Maria Isabel Rodriguez, Mary E Gaffield, Leo Han, Aaron B Caughey

**Affiliations:** a Department of Obstetrics and Gynecology, Oregon Health and Science University, Portland, OR, USA.; b Department of Reproductive Health and Research, World Health Organization, Geneva, Switzerland.

## Abstract

**Objective:**

The association between increased risk of HIV acquisition and use of progestin-only injectables (POIs) is controversial. We sought to compare the competing risks of maternal mortality and HIV acquisition with use of POIs using updated data on this association and considering an expanded number of African countries.

**Methods:**

We designed a decision-analytic model to compare the benefits and risks of POIs on the competing risks of maternal mortality and HIV acquisition on life expectancy for women in 9 African countries. For the purposes of this analysis, we assumed that POIs were associated with an increased risk of HIV acquisition (hazards ratio of 1.4). Our primary outcome was life-years and the population was women of reproductive age (15–49 years) in these countries, who did not have HIV infection and were not currently planning a pregnancy. Probabilities for each variable included in the model, such as HIV incidence, access to antiretroviral therapy, and contraceptive prevalence, were obtained from the literature. Univariate and multivariate sensitivity analyses were performed to check model assumptions and explore how uncertainty in estimates would affect the model results.

**Results:**

In all countries, discontinuation of POIs without replacement with an equally effective contraceptive method would result in decreased life expectancy due to a significant increase in maternal deaths. While the removal of POIs from the market would result in the prevention of some new cases of HIV, the life-years gained from this are mitigated due to the marked increase in neonatal HIV cases and maternal mortality with associated life-years lost. In all countries, except South Africa, typical-use contraceptive failure rates with POIs would need to exceed 39%, and more than half of women currently using POIs would have to switch to another effective method, for the removal of POIs to demonstrate an increase in total life-years.

**Conclusion:**

Women living in sub-Saharan Africa cope with both high rates of HIV infection and high rates of pregnancy-related maternal death relative to the rest of the world. Based on the most current estimates, our model suggests that removal of POI contraception from the market without effective and acceptable contraception replacement would have a net negative effect on maternal health, life expectancy, and mortality under a variety of scenarios.

## Abstract (precis)

Our model suggests that removing progestin-only injectables in Africa would have a net negative effect on maternal health, life expectancy, and mortality under a variety of scenarios.

## INTRODUCTION

The global community has made a commitment to reach 3 key milestones by 2020 to prevent and treat HIV: (1) reduce new HIV infections to fewer than 500,000 globally; (2) decrease AIDS-related deaths to fewer than 500,000 globally, and (3) eliminate HIV-related stigma and discrimination.^1^ While marked improvements have been made in reducing AIDS-related deaths through improved access to therapy, efforts to reduce new HIV infections have stagnated.^1^ Prevention efforts have been limited by inconsistent usage of barrier methods (male or female condoms), lower-than-expected uptake of male circumcision, and slow implementation and uptake of preexposure prophylaxis.^2^^–^^5^ Africa continues to be the area of the world most affected by the HIV epidemic, and women are at particularly high risk of HIV infection.^1^ In fact, the factors that affect HIV infection acquisition disproportionately impact women of reproductive age in sub-Saharan Africa.

Risk of HIV acquisition, especially in sub-Saharan Africa, must be considered within the context of maternal mortality. Sub-Saharan African countries experience the highest maternal mortality rates in the world, accounting for two-thirds of the world's maternal deaths.^6^ Use of contraception plays a critical role in preventing maternal deaths by allowing women to delay early childbearing, limit childbearing, and avoid unintended pregnancies and subsequent unsafe abortions.^7^ However, there is high unmet need for contraceptive services across Africa,^8^^,^^9^ with only 22% of married women using a modern method of contraception.^8^^,^^10^ As a continent, Africa has the highest rates of unintended pregnancy annually, with an estimated 35% of all pregnancies unintended.^11^ High rates of unintended pregnancy are of particular concern in Africa, where acesss to safe abortion services is highly restricted.^11^ Between one-quarter to one-half of all unintended pregnancies end in abortion, and nearly all (>98%) abortions in Africa are unsafe.^12^^,^^13^

Risk of HIV acquisition, especially in sub-Saharan Africa, must be considered within the context of maternal mortality.

Multiple challenges exist to accessing the most effective forms of modern, reversible contraception, including the intrauterine device (IUD), the progestin-only implant, and progestin-only injectables (POIs), in Africa and other areas. With perfect use, tthese methods are over 99% effective in preventing unintended pregnancy.^14^ Key barriers to accessing these methods include commodity stock-outs, workforce shortages, and differences in acceptability of the methods. Insertion of the IUD and implant requires specialized training, whereas POIs can be provided safely by community health workers. Currently POIs are the predominant method of contraception used across sub-Saharan Africa, accounting for 43% of modern contraceptive methods used.^15^

Progestin-only injectables are currently the predominant contraceptive method used across sub-Saharan Africa.

Within this context, data on the potential association between the use of POIs and acquisition of HIV has generated considerable attention and controversy.^16^ Prior data on the relationship between HIV acquisition and use of hormonal contraceptives has been mixed, with some studies showing a protective effect and others showing an increased risk.^17^^–^^26^ Few studies have found a statistically significant association, and interpretation of the data has been challenging due to important limitations in the methodology of existing studies. A key limitation has been significant variation in controlling for potential confounders. These potential confounders include pregnancy, coital frequency, condom usage, marital status, transactional sex, and percentage of participants at baseline in the comparison group using nonhormonal contraceptives (≤10% versus >10%). No randomized trial or definitive data exist to guid health care decisions. The World Health Organization (WHO) has followed the evidence closely to provide guidance on use of different types of contraceptives among women at increased risk for HIV acquisition.^16^^,^^27^

A 2016 meta-analysis provides an updated assessment of the potential increased risk of HIV acquisition associated with use of hormonal contraception.^28^ While data for implants is limited, data for all hormonal contraceptive methods are largely reassuring, with the exception of depot medroxyprogesterone acetate (DMPA), a commonly used POI. While confounding remains an important consideration when assessing the data on DMPA and risk of HIV acquisition, with only observational data available, the updated meta-analysis from 2016 suggests a possible stronger and consistent signal of an increased risk across the summarized studies. If the association observed in these pooled studies is causal, the magnitude is estimated at a hazards ratio (HR) of 1.4 (95% confidence interval [CI], 1.23 to 1.59).^28^

An updated meta-analysis from 2016 suggests a possible increased risk of HIV acquisition with use of DMPA injectables.

These issues ares of considerable public health importance globally, and represent a significant challenge for women, health care providers, program managers, and policy makers. This dilemma is most pressing in sub-Saharan Africa, where both rates of HIV acquisition and maternal mortality are the highest. In the absence of definitive studies, we sought to explore the potential impact of changing family programs and policy in response to the possible increased risk of HIV acquisition associated with POI use. Specifically, we wanted to understand how the overall balance between maternal mortality and HIV acquisition would shift if contraception policies resulted in the removal of POIs from countries' method mix. In the absence of definitive causality data, and the competing demands placed on reproductive health programs, decision analysis can provide guidance in weighing the associated risks and benefits in differing contexts. We sought to build on previous work by incorporating updated estimates for the risk of HIV acquisition with POI use and for maternal mortality.^29^^–^^31^ Our model considers an expanded range of African countries and accounts for variation in HIV incidence, availability of alternative forms of contraception, access to safe abortion, and average life expectancy in each setting.

Our model sought to understand how the overall balance between maternal mortality and HIV acquisition would shift if progestin-only injectables were removed from the method mix.

## METHODS

We designed a decision-analytic model to compare the use the of POIs and their competing risks of maternal mortality and HIV acquisition on life expectancy, or life-years, for women in 9 African countries (Burkina Faso, Chad, Democratic Republic of the Congo, Kenya, Senegal, South Africa, Malawi, Tanzania, and Uganda). Decision analysis allows a stepwise comparison of probabilities and outcomes associated with differing policies.^32^^–^^34^

For the purposes of this analysis, we assumed that POIs were associated with an increased risk of HIV acquisition. We used the reported magnitude of association of 1.4 from the pooled meta-analysis as our base estimate.^28^ HIV incidence, access to antiretroviral therapy (ART), maternal mortality, and contraceptive prevalence vary widely within Africa.^6^^,^^8^^,^^35^^,^^36^ We therefore selected 9 African countries across distinct subregions and specifically included South Africa, a country where previous modeling has demonstrated that the balance between benefit and harm is most nuanced.^29^^,^^30^ The population of focus was women of reproductive age in these countries, who did not have HIV and who were not currently planning a pregnancy.

We included 9 African countries across distinct subregions in the model.

The model begins by comparing a scenario where POIs are available (i.e., current policy) with a scenario where POIs have been eliminated (Figure). POIs include both DMPA and norethisterone enanthate (NET-EN). The model focuses on use of modern, reversible methods; it does not consider use of permanent methods. In the model, a woman may choose to use (1) nonhormonal contraception (copper IUD), (2) hormonal contraception (POIs, implant, or combined oral contraceptives [COCs]), or (3) no method (including traditional methods).

**FIGURE:**
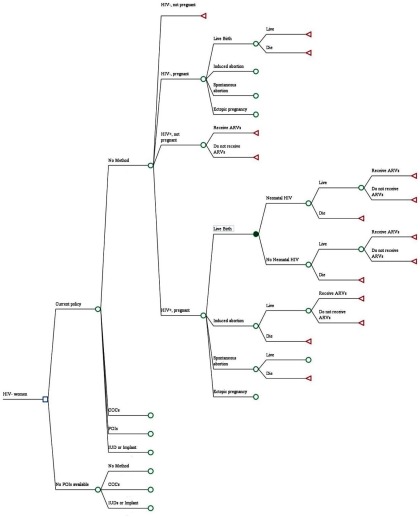
Decision Analysis Model Abbreviations: ARVs, antiretrovirals; COCs, combined oral contraceptives; IUD, intrauterine device; POI, progestin-only injectable. All branches are followed to the same outcome of life expectancy; truncated here for clarity. Red triangles represent terminal nodes while green circles represent decision nodes. When calculating the number of HIV cases averted if no POIs were available, we considered 2 scenarios for women currently using POIs: (1) they all switch to no method, and (2) they switch to another reversible modern method (COCs, an IUD, or an implant).

While consistent condom usage is known to decrease HIV transmission, reported use remains low.^10^ Our baseline assumption was that condom usage was the same between groups. All pathways are followed over 1 year. Our primary outcome was life-years. We estimated the impact POIs have on life-years by calculating the difference between the number of years we would expect in the absence of POI use compared with what is observed with current POI use. We assumed an average maternal age of 25 at the time of birth, and followed women to the average life expectancy for each country. Life-years were predominantly impacted in 2 ways: maternal mortality and HIV acquisition. Maternal deaths were assumed to occur at the beginning of the model sequence. A woman dying in childbirth would thus not contribute any life-years. HIV acquisition reduced life-years as well. Evidence supports that with ART, even in low-resource settings, life expectancy is greatly improved.^37^^,^^38^ Based on these data, we assumed that for women diagnosed with HIV, access to ART would lead to a 25% reduction in life expectancy compared with a woman from the same country without HIV.^38^ For a woman diagnosed with HIV and unable to receive ART, the model assumes that her life expectancy would be reduced by 75%. Secondary outcomes included maternal mortality and new cases of HIV annually (both maternal and neonatal). A standard discount rate of 3% was applied to life expectancy calculations to adjust for inflation.^39^

We estimated the impact progestin-only injectables have on life-years by calculating the difference between the number of years expected in the absence of POI use compared with what is observed with current POI use.

We searched the literature for probabilities for each model variable and data specific to sexually active women of reproductive age (15–49 years) in each country (Table 1). Incidence of HIV by country and country-level probabilities of obtaining ART were obtained from the Joint United Nations Programme on HIV/AIDS (UNAIDS).^36^ Use of ART among women with HIV was assumed to decrease maternal-to-child transmission of HIV from 25% to 2%.^40^

**TABLE 1.** Model Inputs

Variable	All Regions
Increase in HIV acquisition with POI use^28^	40%
Average maternal age at time of birth	25 years
Reduction in life expectancy for women with HIV on ART^38^	25%
Reduction in life expectancy for women with HIV not on ART^38^	75%
Probability of maternal-to-child transmission of HIV if ARVs used in labor^40^	2%
Probability of maternal-to-child transmission of HIV if ARVs not used in labor^40^	25%
Standard discount rate^a^^,^^39^	3%

Regional Inputs									
	Central Africa		Eastern Africa			Southern Africa		Western Africa	
	Chad	DRC	Kenya	Tanzania	Uganda	Malawi	South Africa	Burkina Faso	Senegal
**Life expectancy (years)**^41^	51.5	58.7	61.6	65.0	58.5	62.7	57.2	58.2	66.4
**HIV**^36^									
HIV incidence	.001	.0003	.003	.002	.005	.004	.015	.0005	.0001
Probability of ARVs in labor	.46	.67	.74	.86	.97	.79	.99	.89	.36
Probability of accessing ARVs	.36	.33	.59	.57	.57	.61	.48	.54	.40
**Contraceptive use**^8^^,^^9^									
POIs	.09	.012	.14	.087	.14	.32	.28	.061	.052
COCs	.005	.007	.07	.06	.03	.02	.05	.02	.041
Implant	.00	.007	.016	.005	.025	.09	.00	.08	.012
IUD	.00	.002	.023	.002	.005	.01	.01	.001	.006
Sterilization	.001	.007	.041	.041	.027	.10	.14	.002	.002
**Unmet need for contraception**^b^^,^^8^	.22	.28	.29	.26	.35	.38	.13	.25	.31
**Maternal mortality**^6^									
Live birth^c^	856	693	510	546^d^	343	634	138	371	315
Unsafe abortion^d^^,^^e^	80	80	100	100	100	40	40	80	80
**Pregnancy outcomes (per 100 women)**^13^									
Live birth	.77	.77	.76	.76	.76	.66	.66	.78	.78
Induced abortion	.13	.13	.14	.14	.14	.24	.24	.12	.12
Spontaneous abortion	.09	.09	.09	.09	.09	.09	.09	.09	.09
Ectopic pregnancy	.01	.01	.01	.01	.01	.01	.01	.01	.01

Abbreviations: ART, antiretroviral therapy; ARVs, antiretrovirals; COC, combined oral contraceptive; DRC, Democratic Republic of the Congo; IUD, intrauterine device; POI, progestin-only injectable.

a The standard discount rate is routinely used in decision analysis to account for the fact that goods (dollars, health) are not as valuable in the future as they are in the present. Anywhere between 1.5% and 5% is considered a reasonable rate to discount health outcomes.^37^

b The number of women of reproductive age who are married or living with a partner who are fecund, are not using contraception, and report that they do not want any more children or wish to delay their next pregnancy, divided by the number of women of reproductive age who are married or living with a partner.

c 2015 WHO estimates of maternal mortality ratio (per 100,000 live births).

d Country-level data were not available and so regional estimates were used.

e 2008 WHO unsafe abortion rates (per 1,000 women ages 15–44 years).

Data on contraceptive prevalence and distribution of methods used were obtained for each country from the United Nations.^42^ Data on typical-use contraceptive failure rates were obtained from an analysis of Demographic Health Survey data.^9^ We relied on WHO estimates for maternal mortality rates for both live births and induced abortions.^6^ The probability of a pregnancy ending in induced abortion was obtained for each country from Sedgh and colleagues.^12^^,^^13^

Average life expectancy by country was obtained from the United Nations.^41^ We assumed that POIs are associated with an increased risk of HIV acquisition, and used the summary odds ratio from the published meta-analysis as our baseline estimate (HR 1.4, 95% CI, 1.23 to 1.54).^28^ We calculated the number of cases of HIV averted as the total number of new cases of HIV (maternal and neonatal) that would be expected in each country both with and without POI use. When calculating expected cases without POI use, we considered 2 scenarios: (1) all women currently using POIs switched to no method, and (2) all women currently using POIs switched to another reversible modern contraceptive method, either COCs, an IUD, or an implant. Cases of HIV averted were compared with the difference in maternal deaths we would expect both with POI use and without. In order to assess the impact on mortality, we used the outcome value of “life-years” and calculated it across both scenarios.

The robustness of the model was evaluated with both univariate and multivariate sensitivity analyses to explore how changes in parameters such as HIV incidence, maternal mortality, and contraceptive prevalence could affect the observed results. Every variable was investigated for a threshold value. A threshold value marks the point at which a change in a variable's value would affect the model's conclusion. We ranged each variable from 50% to 200% of the baseline estimate to assess how uncertainty in the estimates would affect our model's conclusions. Two-way sensitivity analysis was performed on all variables with threshold values and other key variables, such as the efficacy of contraceptive methods and availability of IUDs or implants. We performed a Monte Carlo simulation using 1,000 trials to evaluate how simultaneous multivariable changes would affect outcomes. The Monte Carlo simulation enabled variation of all probability estimates simultaneously by sampling distributions around the baseline estimate.

The model was evaluated with both univariate and multivariate sensitivity analyses to explore how changes in parameters could affect the observed results.

### Ethics Approval

As a decision analysis using publicly available information, this project was exempt from review by an Institutional Review Board.

## RESULTS

### Replacement of POIs With No Method Use

In the model, discontinuation of POIs without replacement with an equally effective reversible contraceptive method would result in decreased life-years in each country due to a significant increase in maternal deaths from unintended pregnancy. On average, we estimate that 9,000 life-years per 100,000 women would be lost across all countries. While the policy change would result in the prevention of some new cases of maternal HIV in the model, the life-years gained from this are mitigated due to the marked increase in neonatal HIV cases and maternal mortality with associated life-years lost (Table 2). The increased number of neonatal HIV cases and increased maternal mortality are the result of increases in births among women with HIV infection since they are no longer using POIs and thus are lacking contraceptive protection. This analysis takes into account the probability of provision of ARVs during labor (which can reduce mother-to-child transmission of HIV to the newborn).

**TABLE 2.** Comparison of Baseline Scenario of Current POI Use With the Scenario of Eliminating POIs From the Market and All POI Users Switching to No Method (per 100,000 Women)

	Baseline	Remove POIs	Difference
**CENTRAL AFRICA**			
**Chad**			
Change in life-years			−9000 life-years lost
New HIV cases (total)	171	155	−16 HIV cases
New maternal HIV cases	161	134	−27 HIV cases
New neonatal HIV cases	10	21	+11 HIV cases
Maternal deaths	363	755	+391 maternal deaths
**Democratic Republic of the Congo**			
Change in life-years			−6600 life-years lost
New HIV cases (total)	422	409	−13 HIV cases
New maternal HIV cases	390	342	−48 HIV cases
New neonatal HIV cases	32	67	+35 HIV cases
Maternal deaths	190	500	+310 maternal deaths
**EASTERN AFRICA**			
**Kenya**			
Change in life-years			−6600 life-years lost
New HIV cases (total)	423	409	−14 HIV cases
New maternal HIV cases	398	349	−49 HIV cases
New neonatal HIV cases	25	60	+35 HIV cases
Maternal deaths	190	551	+341 maternal deaths
**Tanzania**			
Change in life-years			−7000 life-years lost
New HIV cases (total)	267	259	−8 HIV cases
New maternal HIV cases	248	217	−31 HIV cases
New neonatal HIV cases	19	42	+23 HIV cases
Maternal deaths	201	406	+210 maternal deaths
**Uganda**			
Change in life-years			−5000 life-years lost
New HIV cases (total)	622	592	−30 HIV cases
New maternal HIV cases	581	509	−72 HIV cases
New neonatal HIV cases	41	83	+ 42 HIV cases
Maternal deaths	196	406	+210 maternal deaths
**SOUTHERN AFRICA**			
**Malawi**			
Change in life-years			−7500 life-years lost
New HIV cases (total)	489	473	−16 HIV cases
New maternal HIV cases	456	400	−56 HIV cases
New neonatal HIV cases	33	73	+40 HIV cases
Maternal deaths	252	565	+313 maternal deaths
**South Africa**			
Change in life-years			−1000 life-years lost
New HIV cases (total)	1774	1771	−3 HIV cases
New maternal HIV cases	1624	1507	−117 HIV cases
New neonatal HIV cases	150	264	+114 HIV cases
Maternal deaths	186	332	+146 maternal deaths
**WESTERN AFRICA**			
**Burkina Faso**			
Change in life-year			−4700 life-years lost
New HIV cases (total)	47	46	−1 HIV case
New maternal HIV cases	43	38	−5 HIV cases
New neonatal HIV cases	4	8	+ 4 HIV cases
Maternal deaths	172	349	+177 maternal deaths
**Senegal**			
Change in life-years			−4500 life-years lost
New HIV cases (total)	13	12	−1 HIV case
New maternal HIV cases	12	10	−2 HIV cases
New neonatal HIV cases	1	2	+1 HIV case
Maternal deaths	147	304	+157 maternal deaths

Abbreviation: POI, progestin-only injectable.

Discontinuation of progestin-only injectables without replacement with an equally effective reversible method would result in decreaed life-years.

Within individual countries, however, large variations are observed. For example, the number of incident HIV infections prevented if POIs were removed from the national formulary ranged from 1 per 100,000 women in Burkina Faso and Senegal to 30 per 100,000 in Uganda (Table 2). However, this action would also be accompanied by more maternal deaths (177 and 157 per 100,000 women in Burkina Faso and Senegal, respectively, and 210 per 100,000 in Uganda) and overall decreases in life expectancy in both countries. Averting new cases of HIV through the removal of POIs would result in increased maternal deaths in all countries; this varied from 146 additional maternal deaths in South Africa to 391 in Chad. This finding persisted across all values of maternal mortality, HIV incidence, and contraceptive failure rates and ranged from baseline to twice the initial estimate. In South Africa, the HIV incidence rate would need to increase to more than .018, or the failure rate of POIs would need to exceed 29.4%, for POI use to not be associated with increased life-years.

Averting new HIV cases through the removal of progrestin-only injectables would result in increased maternal deaths in all countries included in the model.

### Replacement of POIs With the IUD or Implant and Sensitivity Analysis

We then considered the effect of replacing POI use with an IUD or implant. Switching from POIs to an IUD or implant would decrease new HIV cases while maintaining or improving life expectancy. However, these findings assume that nearly all women would transition from a POI to an IUD or implant in order for the removal of POIs from the method mix to result in increased life-years. The threshold value of the percentage of women who would need to switch to an IUD or implant for the removal of POIs to result in such an increase varied by country (Table 3). In South Africa, a country with high HIV incidence and relatively low maternal mortality, the lowest threshold value was observed: at least 15.2% of women would need to transition from POIs to an IUD or implant in order for the removal of POIs to result in increased life-years. A very different situation emerged in Chad, where HIV incidence and access to ART and contraception are all comparatively low, yet maternal mortality is high: 96.9% of women currently using POIs would need to transition to an IUD or implant in order for the removal of POIs to result in an increase in life-years (Table 3).

**TABLE 3.** Sensitivity Analysis Results: At What Threshold Value^a^ Would Removal of POIs Result in an Increase in Life-Years?

Variable	Central Africa		Eastern Africa			Southern Africa		Western Africa	
	Chad	DRC	Kenya	Tanzania	Uganda	Malawi	South Africa	Burkina Faso	Senegal
POI contraceptive failure rate	82.8%	78.7%	79.3%	81.4%	81.1%	81.0%	14.4%	84.2%	84.7%
% of women switching to an equally effective method^b^	96.9%	92.8%	93.5%	96.1%	95.4%	94.9%	15.2%	99.6%	100.0%
Maternal mortality ratio	–^c^	–^c^	–^c^	–^c^	–^c^	–^c^	−23%	–^c^	–^c^

Abbreviation: DRC, Democratic Republic of the Congo.

a A threshold value indicates the value a variable would need to reach or exceed for removal of POIs to result in increased life-years. For example, in South Africa the maternal mortality ratio would need to decrease by 23% for the removal of POIs to be associated with increased life-years, assuming all other variables remain the same.

b Intrauterine device or implant.

c No threshold value exists; across all values of the maternal mortality ratio, removal of POIs results in loss of life-years.

Contraceptive effectiveness rates are thought to vary internationally due to imperfect use and method discontinuation.^43^ We closely examined contraceptive failure rates in our model. Table 3 shows the threshold values for the contraceptive failure rate, or pregnancy rate, of POIs by country. In all countries, except South Africa, the failure rate with POIs would need to be between 78% to 85% for the removal of POIs to be associated with an increase in life-years. In South Africa, the threshold value for contraceptive failure was much lower, at 14.4%.

We performed 2-way sensitivity analyses on probability of method failure with each contraceptive and HIV incidence, as well as probability of switching methods. In all countries, except South Africa, contraceptive failure rates with POIs would need to exceed 39% and more than half of women would have to switch to another method for removal of POIs to demonstrate an increase in total life-years. In South Africa, contraceptive failure rates with POIs would need to exceed 18%, and 45% of women would have to switch to another method, for the removal of POIs to yield an increase in life-years.

In Chad, Kenya, and Uganda, the removal of POIs decreased life-years even if we assumed a 3% incidence of HIV annually and a 40% contraceptive failure rate of POIs. In South Africa, a country where safe abortion is accessible, use of modern contraceptives is relatively high, and access to ART is widespread, a lower threshold is identified: if the failure rate with POIs exceeds 29%, at the current HIV incidence of 1.5% annually, the removal of POIs would increase life-expectancy.

Monte Carlo simulations, which sample the distribution around each input of the model allowing for simultaneous consideration of uncertainty, were performed for each country. These analyses revealed our results to be robust. In every country, except for South Africa, the use of POIs was associated with increased life-years in nearly all simulations (range 98% to 100%). In South Africa, however, POI use was the preferred strategy in only 81% of trials.

## DISCUSSION

Women living in sub-Saharan Africa cope with both high rates of HIV infection and high rates of pregnancy-related maternal death relative to the rest of the world. Based on the most current data on the possible increased risk of HIV acquisition with POIs and on maternal mortality for 9 diverse sub-Saharan African countries, our model suggests that removal of POI contraception from the market without effective and acceptable contraception replacement would have a net negative effect on maternal health, life expectancy, and mortality. In the base case where POIs are removed without substituting POI use with another effective reversible method, we estimate an average loss of 9,000 life-years per 100,000 women across all countries in this region. Even where another equally effective method, such as IUDs or implants are substituted, in countries with the highest maternal mortality rates, an unrealistically large proportion of women would need to transition to the new method in order to reach net neutral mortality thresholds. In countries with high HIV incidence and a relatively low maternal mortality rate, such as South Africa, the balance of benefits and harms is narrower. Our findings support previous research.^29^^,^^30^^,^^31^ Other studies modeling the withdrawal of POIs from the market found a similar increase in maternal mortality, as well as a large increase in unintended pregnancy. These simulations highlight the critical role contraception provision plays in preserving maternal life in Africa and the perils faced by women in this region.

Rapid removal of progestin-only injectables from the method mix could lead to the unintended consequence of greatly increasing maternal morbidity and moratlity.

In countries with the highest maternal mortality rates, an unrealistically large proportion of the women would need to transition from progestin-only injectables to another effective method in order to reach net neutral mortality thresholds.

The implications of changes to contraceptive policies and programs surrounding POIs are significant, particularly in sub-Saharan Africa. Most countries with high HIV prevalence offer few contraceptive options for women to choose from; injectables such as DMPA and NET-EN are familiar and widely used methods. With 28.8% of the contraceptive users in sub-Saharan Africa choosing POIs,^42^ a rapid removal of POIs from the method mix could lead to the unintended consequence of greatly increasing maternal morbidity and mortality. Even if the contraceptive method mix were to broaden in this region, significant efforts would need to be made to ensure women found the new methods to be both accessible and acceptable.

The impact of family planning provision has been recognized by policy and program organizations as critical to improving health outcomes worldwide. Moreover, family planning has been identified as a key accelerator to achieving the Sustainable Development Goals including impacting maternal, child, and adolescent health, shaping regional economic development, and progressing human rights and gender equity.^44^ These far-reaching implications of family planning mean that implementing new contraceptive policy must take into account the broader picture. Even our analysis, which accounts for maternal mortality, likely underestimates the health and socioeconomic implications of reduced access to widely used contraceptives.

### Limitations

We must caution that our analysis uses data from a research area of continued controversy and debate. Our model assumes that the observed association between POI usage and HIV acquisition is real, and our base analysis used the pooled risk ratio (HR 1.4) from this presumed association. However, this association is not consistently seen in previous studies and more definitive studies are still in process.

Another limitation to our analysis is that we relied on data from the UN to estimate contraceptive use rates and HIV incidence. These data, despite being the best available, are notoriously difficult to estimate as contraceptive use is not routinely collected or reported to national and global monitoring bodies. However, our sensitivity analysis used wide ranges to account for uncertain inputs. Even in the setting of much higher HIV incidence or contraceptive failure, our conclusions remained the same.

Finally, our models also assume condom usage and coital frequency to be identical across all groups. If real-life differences between groups exist, this could impact both the risk of HIV acquisition and the rate of pregnancy.

## CONCLUSION

The important link between the HIV epidemic, contraception provision, and maternal health was long established before controversy on POI usage and HIV acquisition emerged. HIV infection remains a large cause of maternal death in sub-Saharan Africa and the availability and usage of barrier methods and dual protection systems remain critical to prevent the spread of HIV.^45^ Furthermore, ART and preexposure prophylaxis both play important roles in HIV transmission and acquisition and reproductive health. Our model found that removal of POIs from the market without effective and acceptable contraception replacement would have a net negative effect on maternal health, life expectancy, and mortality, and this persisted under a variety of modeled scenarios. Policy and programmatic decisions about the role of POIs in family planning programs must therefore be made cautiously, with continued recognition of the interconnectedness of these health issues.

## Notes

**Peer Reviewed**

**Funding:** World Health Organization, Department of Reproductive Health and Research.
